# Molecular analysis of pediatric CNS-PNET revealed nosologic heterogeneity and potent diagnostic markers for CNS neuroblastoma with *FOXR2*-activation

**DOI:** 10.1186/s40478-021-01118-5

**Published:** 2021-02-03

**Authors:** Andrey Korshunov, Konstantin Okonechnikov, Felix Schmitt-Hoffner, Marina Ryzhova, Felix Sahm, Damian Stichel, Daniel Schrimpf, David E. Reuss, Philipp Sievers, Abigail Kora Suwala, Ella Kumirova, Olga Zheludkova, Andrey Golanov, David T. W. Jones, Stefan M. Pfister, Marcel Kool, Andreas von Deimling

**Affiliations:** 1 Clinical Cooperation Unit Neuropathology (G380), German Cancer Research Center (DKFZ), Im Neuenheimer Feld 280, 69120 Heidelberg, Germany; 2 German Cancer Consortium (DKTK), Heidelberg, Germany; 3 Department of Neuropathology, Heidelberg University Hospital, Heidelberg, Germany; 4 Hopp Children’s Cancer Center Heidelberg (KiTZ), Heidelberg, Germany; 5 Division of Pediatric Neurooncology, German Cancer Research Center (DKFZ) and German Cancer Consortium (DKTK), Heidelberg, Germany; 6 Department of Neuropathology, NN Burdenko Neurosurgical Institute, Moscow, Russia; 7 Department of Neuro-Oncology, Russian Scientific Center of Radiology, Moscow, Russia; 8 Department of Neuroradiology, NN Burdenko Neurosurgical Institute, Moscow, Russia; 9 Division of Molecular Genetics, German Cancer Research Center (DKFZ) and German Cancer Consortium (DKTK), Heidelberg, Germany; 10 Pediatric Glioma Research Group, German Cancer Research Center (DKFZ), Heidelberg, Germany; 11 Department of Pediatric Hematology and Oncology, Heidelberg University Hospital, Heidelberg, Germany; 12 Princess Máxima Center for Pediatric Oncology, Utrecht, The Netherlands

**Keywords:** CNS-PNET, Neuroblastoma, *FOXR2*-activation, SOX10

## Abstract

Primitive neuroectodermal tumors of the central nervous system (CNS-PNETs) are highly malignant neoplasms posing diagnostic challenge due to a lack of defining molecular markers. CNS neuroblastoma with forkhead box R2 (*FOXR2*) activation (CNS_NBL) emerged as a distinct pediatric brain tumor entity from a pool previously diagnosed as primitive neuroectodermal tumors of the central nervous system (CNS-PNETs). Current standard of identifying CNS_NBL relies on molecular analysis. We set out to establish immunohistochemical markers allowing safely distinguishing CNS_NBL from morphological mimics. To this aim we analyzed a series of 84 brain tumors institutionally diagnosed as CNS-PNET. As expected, epigenetic analysis revealed different methylation groups corresponding to the (1) CNS-NBL (24%), (2) glioblastoma IDH wild-type subclass H3.3 G34 (26%), (3) glioblastoma IDH wild-type subclass MYCN (21%) and (4) ependymoma with *RELA_C11orf95* fusion (29%) entities. Transcriptome analysis of this series revealed a set of differentially expressed genes distinguishing CNS_NBL from its mimics. Based on RNA-sequencing data we established *SOX10* and *ANKRD55* expression as genes discriminating CNS_NBL from other tumors exhibiting CNS-PNET. Immunohistochemical detection of combined expression of SOX10 and ANKRD55 clearly identifies CNS_NBL discriminating them to other hemispheric CNS neoplasms harboring “PNET-like” microscopic appearance. Owing the rarity of CNS_NBL, a confirmation of the elaborated diagnostic IHC algorithm will be necessary in prospective patient series.

## Introduction

Primitive neuroectodermal tumors of the central nervous system (CNS-PNETs) are highly malignant neoplasms that predominantly affect children and adolescents [2, 3, 7, 8, 10, 18, 24, 27]. Histologically, these tumors are characterized by poorly differentiated or undifferentiated cellular composition with a propensity for dual (i.e. both glial and neuronal) differentiation. The term “PNET” has been introduced to separate such lesions from other malignant brain tumors [24] and has subsequently been included in previous versions of the CNS tumors classifications [27]. However, further advances in brain tumor diagnostics revealed that a considerable fraction of brain tumors traditionally imposing as “PNET” unequivocally belonged to other molecularly established tumor entities [27]. Therefore, the term “PNET” was disbanded for the designation of a distinct brain tumor entity in the WHO classification of brain tumors in 2016 [27]. The “2016 replacement” was the introduction of the designation “other CNS embryonal tumors” used for pooling a heterogeneous set of poorly differentiated neuroepithelial tumors, including the medulloepithelioma, CNS neuroblastoma/ganglioneuroblastoma and CNS embryonal tumor, NOS. This concept has experienced major reshuffling again to be introduced in the upcoming 2020 WHO classification. Seminal work underlying the upcoming WHO classification of embryonal CNS tumors is based on current molecular tumor classification [5, 6]. Sturm et al. [27] analyzed DNA methylation profiles of 323 ‘CNS-PNET’ and demonstrated that these histologically uniform poorly differentiated tumors indeed represent a wide spectrum of molecularly defined diagnostic entities including ETMR, various high-grade gliomas, ependymal, pineal neoplasms, and ATRT, among others. In addition, four novel and previously undetermined molecular entities designated respectively as CNS neuroblastoma with forkhead box R2 “CNS NB-*FOXR2*”, “CNS Ewing sarcoma family tumor with CIC alteration (CNS EFT-CIC),” “CNS high-grade neuroepithelial tumor with MN1 alteration (CNS HGNET-MN1),” and “CNS high-grade neuroepithelial tumor with BCOR alteration (CNS HGNET-BCOR)” were identified and characterized in detail. Among these tumor groups arising from the former PNET commonality, CNS NB-*FOXR2* (CNS_NBL) poses a special diagnostic problem due to its closest resemblance to various undifferentiated CNS neoplasms. In contrast, CNS EFT-*CIC* frequently impose as high-grade gliomas, CNS HGNET-*MN1* often are diagnosed as astroblastoma, and CNS HGNET-*BCOR* exhibit histopathological features resulting in grouping with ependymomas. In the current study, we performed integrative DNA- and RNA-based molecular analysis of a cohort of pediatric brain tumors histologically designated as “CNS-PNET”, which were diagnosed and treated in a single Centre aiming to determine their “institutional” nosologic spectrum, establish molecular diagnostic markers and evaluate the effectiveness of treatment within the different molecularly defined entities.

## Materials and methods

### Patient population

Tissue samples were obtained from 84 patients (age 3–18 years) with the histological diagnosis CNS-PNET according to the 2007 WHO classification of tumors of the central nervous system [27]. All these enrolled samples were diagnosed between 01.01.2000 and 31.12.2016 at the Burdenko Neurosurgical Institute in Moscow and all patients received combined treatment according to the HIT protocol (see Results for details) [7, 17]. All tumors were located in cerebral hemispheres whereas tumors of the pineal region (pineoblastomas) were excluded as well as embryonal tumors with multilayered rosettes (ETMR) and atypical teratoid/rhabdoid tumor (AT/RT) based on LIN28A and INI1 immunohistochemistry. In general, “CNS-PNET” consisted of 0.7% of all CNS neuroepithelial neoplasms and 3.4% of all pediatric CNS tumors diagnosed at the Burdenko Institute within this time period. Informed consent was obtained from all patients´ parents or caregivers. This retrospective study was conducted under the auspices of the Ethics Committee of the Burdenko Neurosurgical Institute (Ethical vote number 563/6-16) and those of the University of Heidelberg, in compliance with the Russian Federation and German rules and regulations of the Health Insurance Portability, and in adherence to the tenets of the Declaration of Helsinki. The follow-up analysis was stalled on 01.06.2020 (the end-point of follow up).

### DNA methylation, targeted next-generation sequencing (NGS) and RNA sequencing

DNA (84) and RNA (53) were extracted from formalin-fixed and paraffin-embedded (FFPE) target tumor tissue samples using the automated Maxwell system (Promega, Madison, WI, USA) [15, 16]. DNA was analyzed using the Illumina Human Methylation 450 k or 850 k/EPIC BeadChip array as described [5, 6, 14, 16, 22, 23]. Briefly, DNA methylation analyses were performed in R version 3.3.0 (R Development Core Team). To enable comparability between the both arrays, we removed all probes not represented on the 450 k array. In total, 428,799 probes were kept for analysis. The t-distributed stochastic neighbor embedding (t-SNE) plots were computed via the R package Rtsne [5, 16, 23]. Copy number profiles were generated using the ‘conumee’ package for R. Using the “Heidelberg brain tumor classifier; v11b4” (www.molecularneuropathology.org;), highly characteristic methylation classes of 84 institutionally diagnosed CNS-PNET were established, for which correlations to the respective brain tumor entities in the WHO classification (“reference tumor set”) were evident [5, 6]. Taking into an account that 32 out of these 84 (38%) CNS-PNET have been included in the previous studies [5, 6, 27], they were excluded from the reference set respectively. Classifier scores with a probability greater 0.9 were taken as indicative for the respective methylation classes [5, 23]. Targeted next-generation sequencing (NGS) with 130 cancer-associated genes was performed using the NextSeq 500 (Illumina) as described [25]. Sequence data were mapped to the reference human genome using the Burrows-Wheeler Aligner and were processed using the publicly available SAM tools. RNA sequencing of 53 samples was performed on a NextSeq 500 (Illumina) as previously described [15, 26]. The reads alignments and identification of low-quality or outlier samples was performed as described [15, 20]. Unsupervised tumor samples comparison was performed with hierarchical clustering, principal component analysis, t-SNE analysis, and based on the selection of the top 250, 500 and 1000 most variable genes with log2 RPKM gene expression normalization. Differential gene expression analysis was performed by comparing one molecular group against the others using DESeq 2 R package (adjusted *p* < 0·05) and gene ontology analysis was done using ClueGO (Cytoscape version 3.4) [4, 15]. Fusion discovery was done based on RNA sequencing data using five independent algorithms [20, 26].

### Immunohistochemistry with SOX10 and ANKRD55 antibodies

Immunohistochemistry (IHC) was conducted on 4-μm thick formalin-fixed, paraffin-embedded (FFPE) tissue sections mounted on adhesive slides followed by drying at 80 °C for 15 min. IHC with synaptophysin, MAP2, Olig2, EMA, and L1CAM was performed as describes previously [21, 27]. Additionally, IHC with monoclonal antibody to SOX10 protein (Thermo Fisher Scientific Inc.; AB_2572892; clone 20B7) and polyclonal antibody to ANKRD55 protein (Novus Biological; NBP2-14719) was also applied. IHC was performed with an automated immunostainer (Benchmark; Ventana XT) using antigen-retrieval protocol CC1 for SOX10 (working dilution of 1:100) and CC2 for ANKRD55 (working dilution of 1:20) with incubation for both at 37 °C for 32 min. Nuclear SOX10 staining was assessed in four intensity degrees (negative: no stained nuclei; low: 1–25%; moderate: 25–74%; and high: 75–100%. Cytoplasmic ANKRD55 immunostaining was also into four intensity degrees, similar to SOX10 evaluation.

### Statistics

The distributions of overall survival (OS) and progression free survival (PFS) were calculated according to the Kaplan–Meier method using the log rank test for significance. PFS was calculated from the date of diagnosis until tumor recurrence or last contact for patients who were free of disease. OS was calculated from the date of diagnosis until death of patient from disease or last contact for patients who were still alive. For multivariate analysis, Cox proportional hazards regression models were used. Estimated hazard ratios are provided with 95% confidence intervals and a *p* value from the Wald test. Tests with a *p* value below 0.05 were considered significant.

## Results

### Pathological characteristics of the institutionally diagnosed “CNS-PNET” cohort

**Fig. 1:**
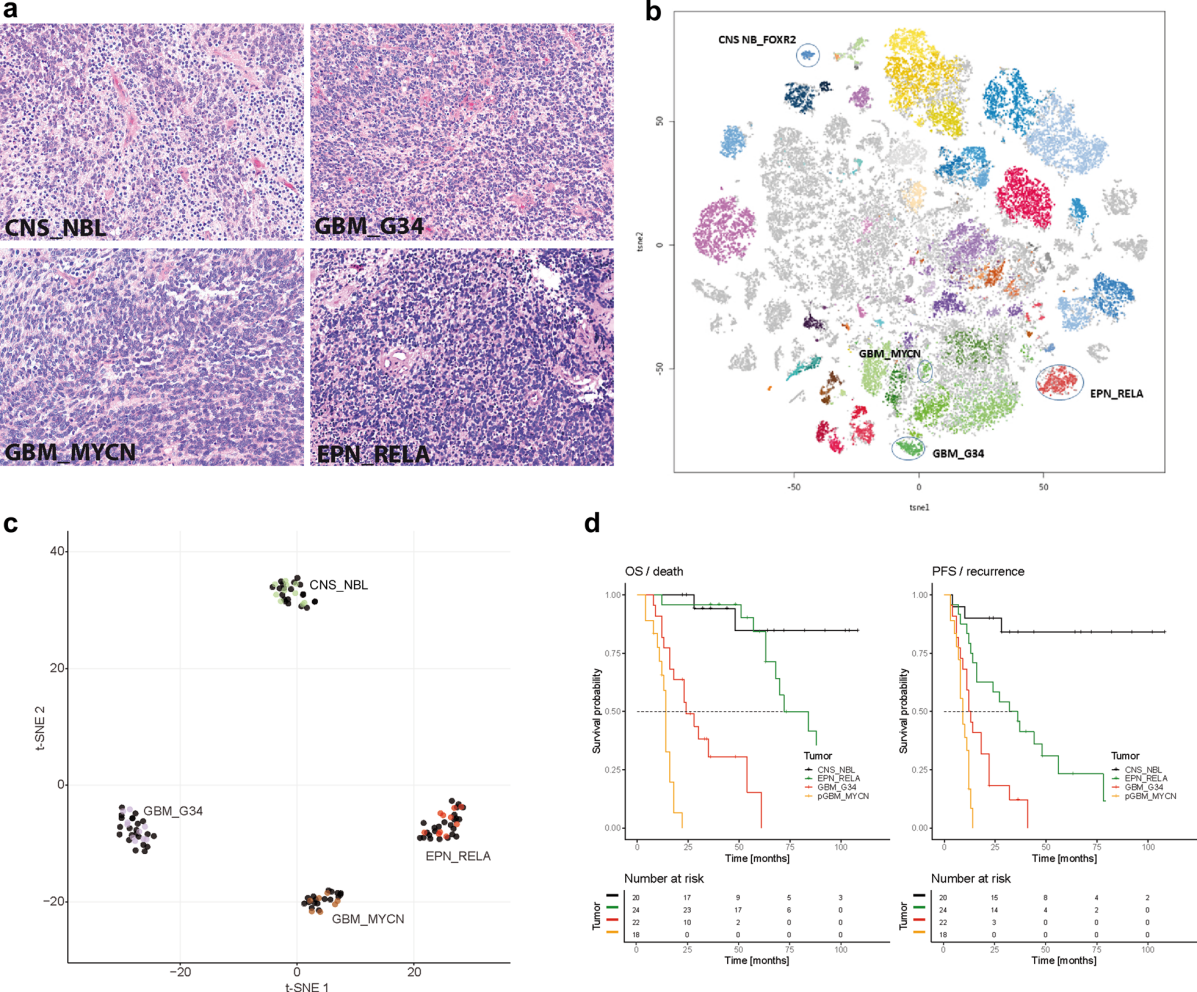
**a**. Pathological similarity of CNS-PNET samples allocated to various molecular groups (The scale bars: 50 μm). **b**. Two-dimensional t-distributed stochastic neighbor embedding (tSNE) analysis of reference set composed of various tumor entities including CNS_NBL, GBM_G34, GBM_MYCN and EPN_RELA cohorts. **c**. tSNE analysis of 84 institutionally diagnosed cPNET (all black spots) revealed distribution their epigenetic profiles among the four reference tumor sets including CNS_NBL (n = 20), GBM_G34 (n = 22), GBM_MYCN (n = 18) and EPN_RELA (n = 24). **d**. Survival analysis for CNS-PNET cohort shows that both overall survival (OS) and progression-free survival (PFS) were significantly better for patients harboring tumors with CNS_NBL molecular signature (black lines), in comparison to GBM_G34 (red lines), GBM_MYCN (orange lines) and EPN_RELA (green lines)

We analyzed 84 samples histologically diagnosed at the central pathological review in Burdenko Institute as “CNS-PNET. Microscopically these poorly differentiated neoplasms were composed of small cells with round to oval nuclei, and high nuclear/cytoplasmic ratio (Fig. 1a). Sometimes collection of cells with large pale nuclei and prominent nucleoli were also found. These tumors revealed frequent mitoses, apoptotic bodies, necrotic foci with various sizes, although pseudopalisading and prominent microvascular proliferation were not identified. No patterns of glial, ependymal or neuronal differentiation were detected besides occasional structures resembling Homer-Wright rosettes in some tumors. Intra-tumoral tumor cell density varied but highly cellular regions were found in all cases. Most of these tumors disclosed a variable positivity for synaptophysin, MAP2, Olig2, EMA,  and L1CAM proteins (see Table 1). We could not determine clear histological features segregating our series into distinct morphological subsets.

**Table 1:** Clinico-pathological characteristics of cPNET

Variable	CNS_NBL (20)	GBM_G34 (22)	GBM_MYCN (18)	EPN_RELA (24)
Age (range)	8 (4–16)	14 (12–18)	8 (3–17)	9 (3–17)
Gender M/F	25%/75%	70%/30%	60%/40%	50%/50%
M 2–3 stage	30%	20%	25%	20%
Resection GTR/NTR	50%/50%	60%/40%	50%/50%	60%/40%
Recurrence	3 (15%)	20 (91%)	17 (94%)	18 (75%)
5-year PFS	80%	0	0	20%
Death	2 (10%)	16 (73%)	16 (89%)	11 (46%)
5 year OS	82%	16%	0	72%
Synaptophysin (pos)	76%	83%	24%	12%
OLIG1 (pos)	78%	22%	56%	10%
MAP2 (pos)	82%	77%	22%	34%
L1CAM (pos)	22%	44%	38%	100%
EMA (dot-like)	18%	34%	47%	58%

### DNA methylation profiling identified four molecular groups of CNS-PNET

Initially, the methylation profiles of this CNS-PNET cohort were investigated by t-SNE analysis. The 84 tumors were compared to a reference set encompassing ca. 68500 tumors (see M&M section; Fig. 1b,c). Our analysis showed that the 84 CNS-PNET samples matched with established methylation classes recognized by the Brain Tumor Classifier [5, 6, 23]: (1) CNS neuroblastoma with *forkhead box R2* (*FOXR2)* activation (CNS_NBL; n = 20; 24%): (2) Glioblastoma, IDH wild-type, subclass H3.3 G34 mutant (GBM_G34; n = 22; 26%); (3) Glioblastoma, IDH wild-type, subclass MYCN (GBM_MYCN; n = 18; 21%), and (4) Ependymoma, with *RELA_C11orf95* fusion (EPN_RELA; n = 24; 29%). We did not determine any significant histopathological differences between these four molecular entities, although some immunohistochemical patterns varied between them (Table 1). In contrast to previous studies, we did not find other newly established CNS-PNET variants and epigenetically “unclassifiable” tumor entities [27].

### Clinico-pathological parameters in molecular groups of CNS-PNET

The basic clinical characteristics of all 84 patients with these four molecularly identified tumor entities are summarized in Table 1. Patients with GBM_G34 were older: median age 14.1 years versus 8.2, 8.4 and 9.2 years for patients with CNS_NBL, GBM_MYCN, and EPN_RELA respectively. Female patients prevailed in the CNS_NBL group (75%), whereas the male:female ratio was almost equal for the three other tumor entities. A similar proportion of patients with these molecular entities disclosed metastatic stage M 2–3 at the time of diagnosis (30%/20%/25%/20%). All patients received primary surgery and a frequency of gross total tumor resections was quite similar for all molecular tumor groups (50%/60%/50%/60%). After surgery, all patients received combined treatment according to the HIT-based protocol [7, 17]: including radiotherapy (craniospinal to 24–36 Gy and tumor bed boost to 55–58 Gy) and HIT-based chemotherapy. Tumor recurrences were observed in three patients (15%) with CNS_NBL and two of them died (10%); 5-year PFS and OS for these patients reached 73% and 84%, respectively (Fig. 1d). Clinical outcomes for the other three molecular groups were significantly worse with more favorable outcomes (but frequent local recurrences) for EPN_RELA (5-year OS—68%) but extremely poor for both GBM_G34 and GBM_MYCN (5-year OS—0%).

### Copy-number alterations and mutational landscapes of CNS-PNET molecular subtypes

The four molecular entities disclosed differences in cytogenetic profiles (Table 2). There were no oncogene amplifications in CNS_NBL, whereas 1q gain (100%), 16q loss (70%) and 17q gain (62%) were most frequent CNVs. GBM_G34 disclosed recurrent amplifications of *PDGFRA* and/or *CCND2* (28% and 23% respectively), and losses of 3q (70%), 4q (70%), and 10q (70%). GBM_MYCN showed a high frequency of *MYCN* amplifications (70%; in combination with *ID2* in 40%), followed by gains of 2p (70%), 7 (60%), and 1q (50%). There were no amplifications in EPN_RELA which harbored rearrangements at 11q13, loss of 9p, and *CDKN2A* homozygous deletions as recurrent CNVs (85%, 45% and 25%).Targeted NGS disclosed recurrent H.3.3 mutations in all 22 GBM_G34 (20 H3.3. G34R and 2 G34V); *TP53* and *ATRX* mutations were also frequent in these tumors (88% and 72% respectively). *TP53* mutations were found in 70% of GBM_MYCN and *TERT* promoter mutations were detected in this group (30%). CNS_NBL and EPN_RELA revealed no recurrent mutations, and *RELA_C11orf95* fusions were not detected with the DNA panel sequencing.

**Table 2:** CNVs and mutations in molecular groups of CNS-PNET

Molecular alterations	CNS_NBL (20)	GBM_G34 (22)	GBM_MYCN (18)	EPN_RELA (24)
*MYCN* amplifications	0	0	70%	0
*PDGFRA* amplification	0	30%	0	0
*CCND2* amplification	0	20%	0	0
1q gain	100%	40%	50%	30%
2p gain	20%	10%	70%	10%
3p loss	60%	10%	10%	0
3q loss	0	70%	10%	0
4q loss	10%	70%	10%	10%
6q loss	40%	0	5%	10%
9p loss	0	40%	10%	45%
10q loss	30%	70%	50%	30%
16q loss	70%	20%	10%	0
17p loss	0	10%	50%	0
17q gain	60%	30%	5%	5%
22q loss	0	15%	15%	30%
Recurrent mutations	No	*G34V/R*—22/22	*TP53*—13/18	No
Recurrent fusions (54)	*FOXR2*—14/14	No	*NBAS*—3/12	*RELA*—20/20

### Transcriptome analysis detected set of differentially expressed genes

**Fig. 2:**
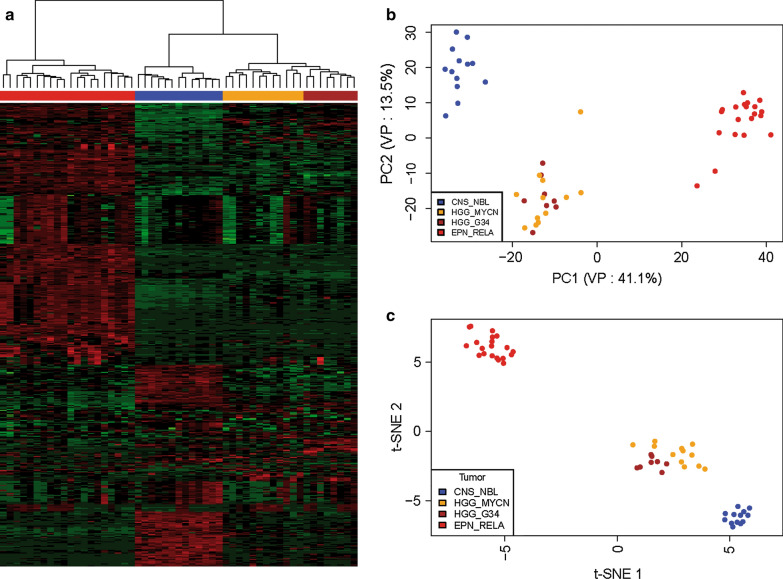
Gene expression profiling data obtained after RNA sequencing of CNS-PNET cohort (n = 54). **a**. Heat-map of unsupervised hierarchical cluster analysis based on 500 differentially expressed genes disclosed that CNS_NBL (blue) were clustered separately from EPN_RELA (red); GBM_G34 (brown), and GBM_MYCN (orange) (Row Z-Scores: green: from 0 to − 6; red: from 0 to 6). Scales of principal component analysis (PCA); **b**. and two-dimensional t-distributed stochastic neighbor embedding (tSNE); **c**. analyses also revealed a separate distribution of CNS_NBL from other molecular groups

In 53 samples we performed RNA sequencing-based analysis in order to assess transcriptional differences between the four “CNS-PNET” molecular groups (13 CNS-NBL, 12 GBM_MYCN, 8 GBM_34 and 20 EPN_RELA). The unsupervised hierarchical clustering (Fig. 2a), principal component (PCA) (Fig. 2b) and t-SNE (Fig. 2c) analyses based on the top 500 most highly variable genes. CNS-NBL and RELA_EPN formed clearly individual clusters while GBM_G34 and GBM_MYCN were also separated each other but composed a common group thus suggesting a somewhat similar transcriptomics.

**Fig. 3:**
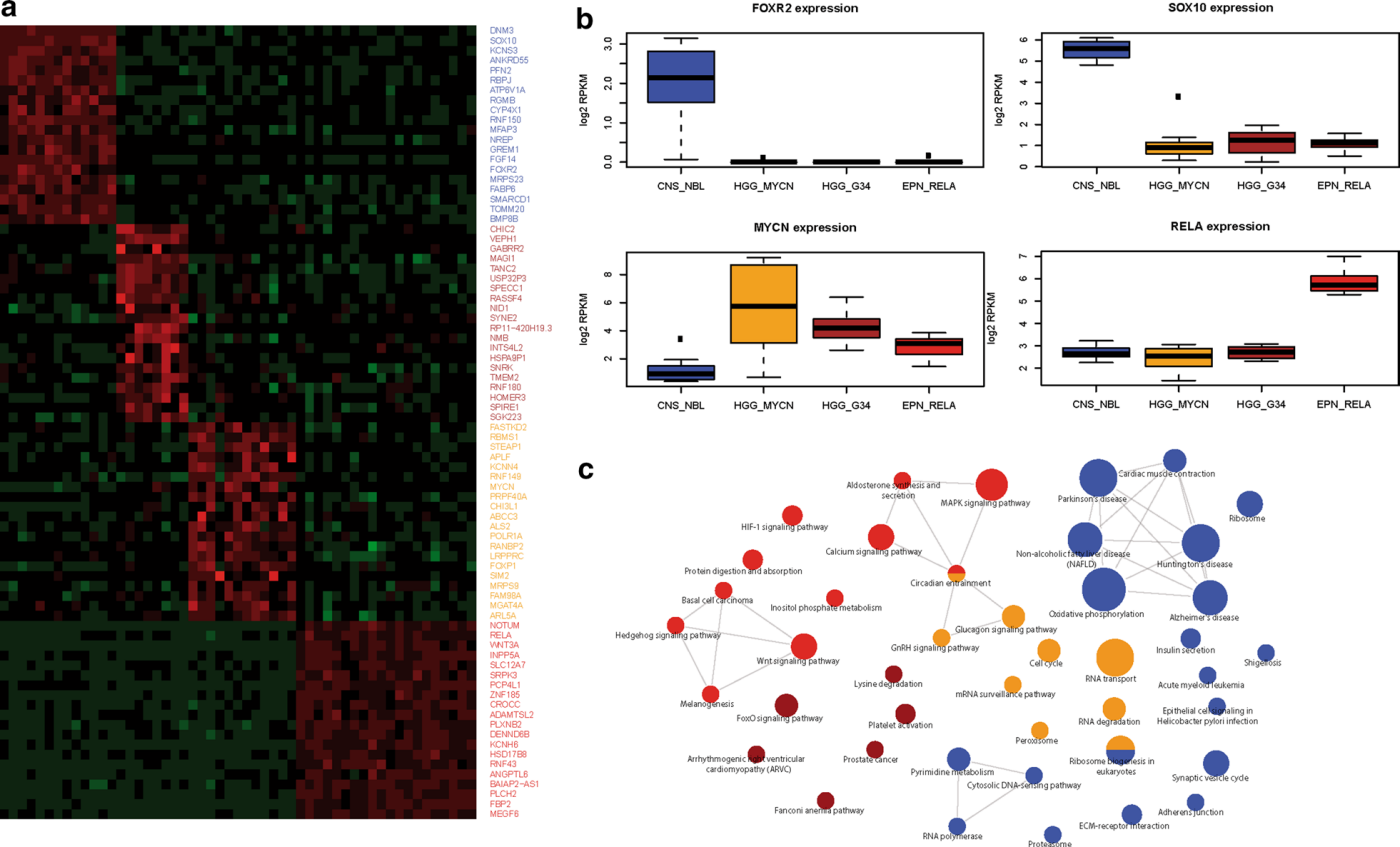
**a**. A set of 30 top most-confident genes differentially overexpressed in CNS_NBL (blue top line); GBM_G34 (brown top line), GBM_MYCN (orange top line), and EPN_RELA (red top line) respectively allows one to discriminate clearly between these tumor cohorts with SOX10 among the top genes for CNS_NBL (Row Z-Scores: green: from 0 to − 6; red: from 0 to 6). **b**. *FOXR2* and *SOX10* expression levels were significantly higher in CNS_NBL (blue boxplots) whereas *MYCN* was overexpressed in GBM_MYCN (orange boxplots) and *RELA*—EPN_RELA (red boxplots) respectively. **c**. Gene Ontology analysis disclosed that CNS_NBL transcriptome signatures (blue circles) were associated with neuronal metabolism, synaptic transmission, and neuroendocrine secretion

Gene fusion transcripts involving the *FOXR2* gene were detected in all 14 CNS_NBL samples and all were accompanied with a high level of gene expression (Fig. 3b). These gene fusions were intergenic duplications involving of *FOXR2* coding sequence (CDS) on chromosome Xp11.21 (Additional file 1: Online Resource; Supplementary Table 1). In three CNS_NBL cases we detected additional translocations involving *FOXR2* and other fusion partners: *LINC00486* at 2p22, and *TM7SF3* at 12p11.23, and *FRMD4A* at 10p11.3. All CNS-PNET with EPN_RELA signature revealed *RELA_C11orf95* fusion at 11q13 (Additional file 1: Online Resource Supplementary Table 2). There were no recurrent fusions in the GBM_G34 and GBM_MYCN molecular groups.

**Fig. 4:**
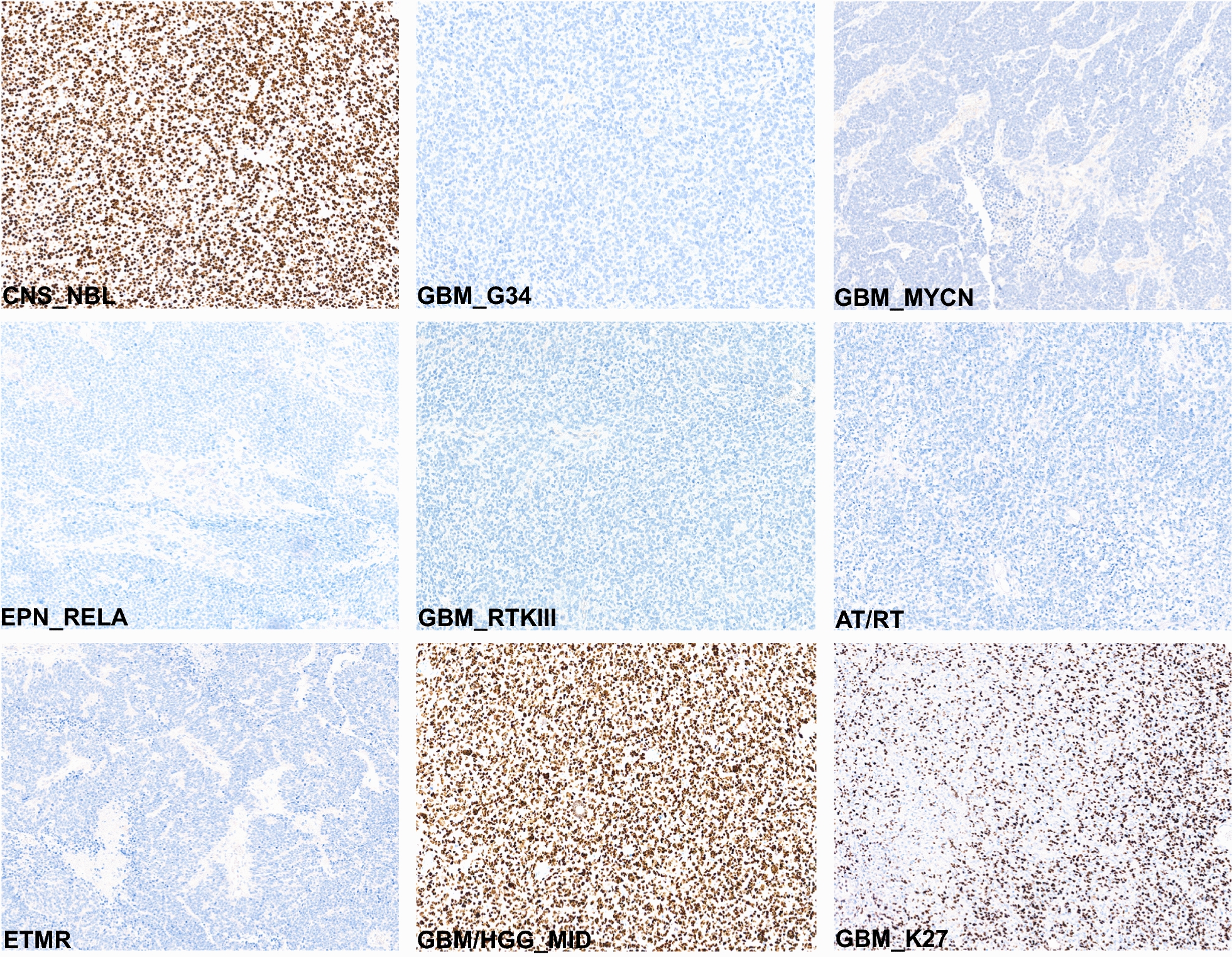
Patterns of SOX10 nuclear protein immunoexpression in various CNS tumors (The scale bars: 50 μm). Intense SOX10 expression was found in all CNS NBL, but also in some subtypes of high-grade gliomas. All embryonal CNS tumors were negative for SOX10, whereas glial neoplasms disclosed various degrees of nuclear immunostaining intensity

Next, expression levels of the gene sets differentially activated between the four outlined molecular entities were compared. A set of the most-confident 30 genes differentially expressed in CNS_NBL (DESeq 2 algorithm; *p* < 0·05) discriminates them from other molecular groups of CNS-PNET with *SOX10* as the top gene in this list (Fig. 3a,b; Additional file 1: Online Resource; Supplementary Table 3). *MYCN* and *RELA* were identified among the overexpressed genes for GBM_MYCN and EPN_RELA respectively, thus confirming a reliability of the expression profiling based on RNA sequencing (Fig. 3b). To delineate characteristic signalling patterns for each of the four cerebral PNET variants, we performed pathway annotation using Gene Ontology analysis (Fig. 3c; Additional file 1: Online Resource Supplementary Table 4). CNS_NBL were enriched with gene sets associated with synaptic transmission, neurotrophic regulation, cell adhesion, and neuroendocrine secretion thus confirming their “neuronal” nature. Transcriptome signatures of GBM_MYCN were characterized by genes involved in RNA replication, ribosome biogenesis and cell cycle activation; GBM_G34—with Fanconi anaemia, FoxO signalling and lysine degradation, and EPN_RELA—with inositol metabolism, WNT, SHH, HIF-1 and basal cell carcinoma signaling pathways (Fig. 4).

### SOX10 is a molecular marker to discriminate of CNS_NBL of CNS-PNET but not of high-grade gliomas

Because of the favorable treatment-dependent outcome of patients with CNS_NBL, we set out to establish robust immunohistochemistry-based marker(s) for their accurate and reproducible pathological diagnosis. We selected *SOX10* for further immunohistochemical (IHC) analysis owing to its discriminative RNA levels for CNS_NBL as detected by subgroup-specific differential gene expression. We stained all 84 molecularly diagnosed CNS-PNET samples with SOX10 monoclonal antibody and also an enlarged set of 263 high-grade pediatric CNS tumors with various histological/molecular diagnoses (for details see Table 3). All 20 CNS NBL samples revealed a high degree of SOX10 nuclear expression, whereas GBM_MYCN, GBM_G34 and EPN_RELA were all estimated as negative samples (Table 3). Stained samples of other embryonal CNS tumors including ATRT, ETMR, HGNET_BCOR, HGNET_MN1 and HGNET_CIC were also completely negative for SOX10. However, some malignant gliomas and especially pediatric high-grade gliomas designated molecularly as H3.3 wt GBM_MID (or RTK1_pGBM) revealed a high degree of SOX10 nuclear expression. Therefore, SOX10 is an appropriate diagnostic marker to discriminate between CNS_NBL and other CNS-PNET/embryonal tumors but it is not sufficient to distinguish them from GBM_MID (sensitivity—92% and specificity 78% for CNS_NBL).

**Table 3:** Immunoexpression of SOX10 protein in various pediatric CNS tumors

Tumor entity	> 75%	25–74%	1–24%	Negative
CNS neuroblastoma with *FOXR2* activation (n = 20)	100%	0	0	0
Pediatric glioblastoma with G34 mutation (n = 25)	0	0	0	100%
Pediatric glioblastoma; MYCN (n = 20)	0	0	0	100%
Supratentorial ependymoma; RELA fusion (n = 40)	0	0	0	100%
Diffuse midline glioma, H3 K27-altered (n = 30)	0	40%	30%	30%
Pediatric glioblastoma, RTKI (pGBM_MID; n = 30)	25%	35%	20%	20%
Pediatric glioblastoma, RTKIII (n = 12)	0	0	0	100%
Adult glioblastoma, IDH-wildtype (n = 40)	0	20%	40%	40%
Adult glioblastoma; IDH-mutant (n = 20)	10%	30%	40%	20%
Anaplastic oligodendroglioma, IDH-mutant (n = 20)	10%	40%	30%	20%
Embryonal tumor with multilayered rosettes (n = 30)	0	0	0	100%
Atypical teratoid/rhabdoid tumor (n = 20)	0	0	0	100%
CNS neuroepithelial tumor; MN1-altered (n = 18)	0	0	0	100%
CNS neuroepithelial tumor; BCOR-altered (n = 8)	0	0	0	100%
CNS neuroepithelial tumor; CIC-altered (n = 5)	0	0	0	100%
Intracranial Ewing sarcoma (n = 4)	0	0	0	100%
Diffuse leptomeningeal glioneuronal tumor (n = 5)	0	0	0	100%

Expression profiling data obtained after Affymetrix-based analysis of independent cohort of various CNS and non-CNS embryonal tumors also showed high expression levels of *SOX10* in CNS_NBL with *FOXR2* alterations as compared to other entities (Additional file 2: Online Resource; Supplementary Figure 1).

### ANKRD55 is a specific marker to discriminate CNS_NBL from pediatric midline HGG

**Fig. 5:**
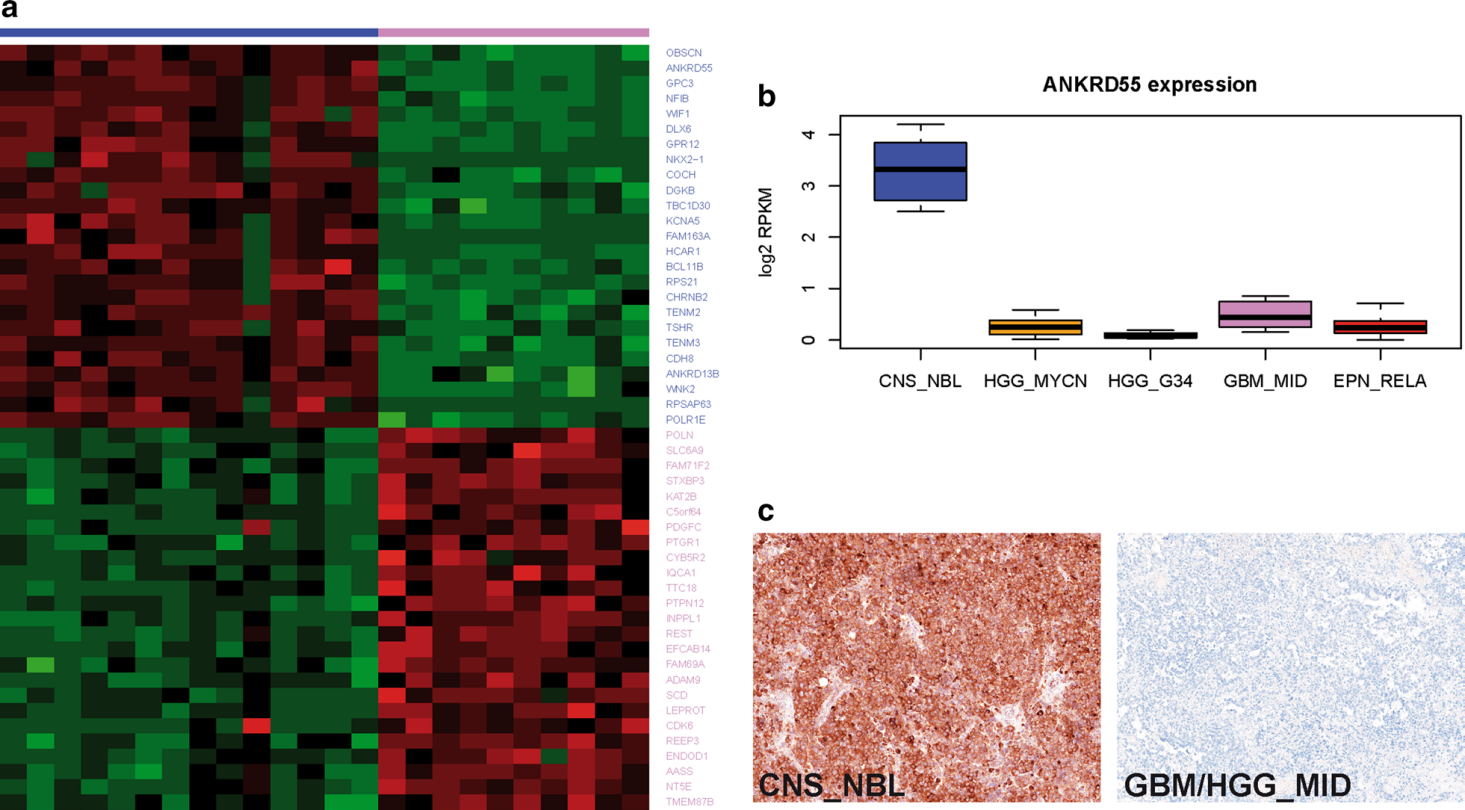
**a**. A set of 25 top most-confident genes differentially overexpressed in CNS_NBL (blue top line) and GBM_MID (pink top line) respectively allows one to discriminate clearly between these tumor cohorts with *ANKRD55* on the top genes for CNS_NBL (Row Z-Scores: green: from 0 to − 6; red: from 0 to 6). **b**. *ANKRD55* expression levels were significantly higher in CNS_NBL (blue boxplots) in comparison to other molecular groups. **c**. Intense ANKRD55 immunostaining was found in CNS_NBL but GBM_MID were negative (The scale bars: 50 μm)

Due to the shortcoming of SOX10 immunostaining to differentiate between CNS_NBL and GBM_MID, we compared expression levels of genes differentially activated in these two entities. Again using the DESeq 2 algorithm we identified *ANKRD55, OBSCN, GPC3, NFIB* and *WIF1* as overexpressed in CNS_NBL whereas expression of *POLN, SLC6A9, FAM71F2* and *STXBP3* was significantly higher in GBM_MID (Fig. 5a). Therefore, we selected *ANKRD55* as a potential diagnostic marker for CNS_NBL taking into account that it was also leading the list of genes differentially expressed between CNS_NBL and other molecular groups of CNS-PNET (Fig. 5b; Additional file 1: Online Resource; Supplementary Table 5). Thus, all 20 CNS_NBL revealed intense ANKRD55 cytoplasmic expression in more than 75% of tumor cells (Fig. 5c; Table 4). All other malignant CNS neoplasms revealed no ANKRD55 staining besides a few ETMR samples which disclosed moderate staining in poorly differentiated areas (sensitivity—97% and specificity 95% for CNS_NBL). We also performed IHC analysis for an independent validation set of 9 tumors diagnosed as CNS_NBL with methylation profiling in other institutions, which all were also intensively stained for both SOX10 and ANKRD55. Thus, an immunohistochemistry targeting SOX10 and ANKRD55 discriminates between CNS_NBL and other malignant pediatric CNS neoplasms (sensitivity—100% and specificity 98% for CNS_NBL).

**Table 4:** Immunoexpression of ANKRD55 protein in various pediatric CNS tumors

Tumor entity	> 75%	25–74%	1–24%	Negative
CNS neuroblastoma with *FOXR2* activation (n = 20)	100%	0	0	0
Pediatric glioblastoma with G34 mutation (n = 25)	0	0	0	100%
Pediatric glioblastoma; MYCN (n = 20)	0	0	0	100%
Supratentorial ependymoma; RELA fusion (n = 40)	0	0	0	100%
Diffuse midline glioma, H3 K27-altered (n = 30)	0	0	0	100%
Pediatric glioblastoma, RTKI (pGBM_MID; n = 30)	0	0	0	100%
Pediatric glioblastoma, RTKIII (n = 12)	0	0	0	100%
Adult glioblastoma, IDH-wildtype (n = 40)	0	0	0	100%
Adult glioblastoma; IDH-mutant (n = 20)	0	0	0	100%
Anaplastic oligodendroglioma, IDH-mutant (n = 20)	0	0	0	100%
Embryonal tumor with multilayered rosettes (n = 30)	0	0	30%	70%
Atypical teratoid/rhabdoid tumor (n = 20)	0	0	0	100%
CNS neuroepithelial tumor; MN1-altered (n = 18)	0	0	0	100%
CNS neuroepithelial tumor; BCOR-altered (n = 8)	0	0	0	100%
CNS neuroepithelial tumor; CIC-altered (n = 5)	0	0	0	100%
Intracranial Ewing sarcoma (n = 4)	0	0	0	100%
Diffuse leptomeningeal glioneuronal tumor (n = 5)	0	0	0	100%

## Discussion

The reception of CNS-PNET as a specific tumor category as well as ways for its diagnosis is a subject of continue controversy [2, 3, 7, 8, 10, 18, 24, 27]. In this study we analyzed a series of 84 poorly differentiated hemispheric neoplasms in children which were diagnosed histologically as CNS-PNET. In line with previous reports, molecular analyses revealed heterogeneity of this tumor cohort encompassing CNS_NBL, RELA_EPN, GBM_G34 and GBM_MYCN. Interestingly, CNS_NBL represented the only embryonal tumor in this cohort, whereas three other molecular groups were “PNET-like” imitations of glial and ependymal descent. Notably, we did not identify three other newly recognized subtypes (CNS HGNET-*MN1,* CNS HGNET-*BCOR* and CNS EFT-*CIC)* in the current institutional series of CNS-PNET [18, 27]. Retrospective analysis of institutional pediatric brain tumor series revealed 12 CNS HGNET-*MN1* diagnosed as anaplastic ependymoma, astroblastoma or HGG, 5 CNS HGNET-*BCOR* diagnosed as anaplastic ependymoma, and 2 CNS EFT-*CIC* diagnosed as HGG (data not shown). All patients in our CNS-PNET series were treated according to the HIT-based protocol of radio-chemotherapy [7, 17]. However, only patients with CNS_NBL exhibited favorable response stressing the necessity of identifying these patients in the pool of CNS_NBL diagnoses. The method of choice for diagnosing CNS_NBL is methylation analysis [5, 6, 14, 22, 23]. However this technology requires resources not accessible on a global scale. Likewise, GBM_G34 and EPN_RELA are readily diagnosed by methylation analysis, however, both entities can be identified recently by targeting the H3.3 G34 mutation or by employing p65-RelA antibodies [9, 21]. In contrast, the unequivocal recognition of GBM_MYCN with non-molecular methods still poses challenges. [18].

Analysis of gene expression data generated for CNS_NBL, GBM_G34, GBM_MYCN and EPN_RELA revealed their specific transcriptional patterns. Highly expressed genes characteristic for CNS_NBL were also explored for their detection by immunohistochemistry on FFPE tissue samples. Two genes among the top scoring at RNA level, *SOX10* and *ANKRD55*, encoded for proteins readily and specifically detected with commercially available antibodies (Tables 3, 4). *SOX10* encodes a transcription factor essential for the development of neural crest, peripheral nervous system and melanocytes [19]. In various neoplasms, SOX10 expression is found in melanomas, epithelial neoplasms, astrocytomas and oligodendrogliomas [1, 12, 13]. *ANKRD55* codes for the Ankyrin repeat domain 55 protein for which function and differentiation specific expression has not been systematically examined [11]. However, some studies reported ANKRD55 expression in cerebral and spinal neurons suggesting its possible role in pathogenesis of multiple sclerosis [11]. Therefore, in contrast to SOX10, ANKRD55 has not been systematically tested for its expression in human brain tumors. Detection of both proteins was not exclusively restricted to CNS_NBL. SOX10 positive IHC was also observed to a lesser degree in GBM_K27 and especially in GBM_MID while ANKRD55 positivity also occurred in ETMR, albeit again with lower intensity. However, the combination of strong immunopositivity for both, SOX10 and ANKRD55 proved highly specific for CNS_NBL, both in institutionally diagnosed CNS-PNET cohort and extended set of malignant pediatric brain tumors.

In conclusion, our findings are in line with previous studies, demonstrating the molecular heterogeneity of pediatric cerebral neoplasms diagnosed as CNS-PNET. Patients with CNS_NBL included in these mixed diagnostic bags benefit from treatment according to HIT protocol. Immunohistochemical detection of combined expression of SOX10 and ANKRD55 clearly identifies CNS_NBL discriminating them to other hemispheric CNS neoplasms harboring “PNET-like” microscopic appearance. Owing the rarity of CNS NBL, a confirmation of the elaborated diagnostic algorithm will be necessary in independent tumor series and prospective clinical trials.

## Supplementary Information

**Additional file 1.**

**Additional file 2.**
