# Supplementary material for: Molecular analysis of pediatric CNS-PNET revealed nosologic heterogeneity and potent diagnostic markers for CNS neuroblastoma with FOXR2-activation
Source: Acta Neuropathol Commun. 2021 Feb 3;9:20. doi: 10.1186/s40478-021-01118-5 (PMC7860633; doi:10.1186/s40478-021-01118-5)
Supplement: Supplementary file 1 — Additional file 1. [file 40478_2021_1118_MOESM1_ESM.docx]

| **Case Number** | **Gene1** | **Gene2** | **Breakpoint_1** | **Breakpoint_2** | **Site_1** | **Site_2** | **Type** |
| --- | --- | --- | --- | --- | --- | --- | --- |
| **CNS_NBL_1** | FOXR2 | FOXR2 | X:55650509 | X:55650921 | CDS | CDS | duplication |
| **CNS_NBL_1** | FOXR2 | PCSK5 | X:55650698 | 9:78862685 | CDS | intron | translocation/5'-5' |
|  |  |  |  |  |  |  |  |
| **CNS_NBL_2** | FOXR2 | FOXR2 | X:55650505 | X:55650819 | CDS | CDS | duplication |
|  | FOXR2 | TM7SF3 | X:55654547 | 12:27143521 | intergenic | CDS | translocation |
| **CNS_NBL_3** | FOXR2 | FOXR2 | X:55650291 | X:55650814 | CDS | CDS | duplication |
|  |  |  |  |  |  |  |  |
| **CNS_NBL_4** | FOXR2 | FOXR2 | X:55650309 | X:55650799 | CDS | CDS | duplication |
|  |  |  |  |  |  |  |  |
| **CNS_NBL_5** | FOXR2 | FOXR2 | X:55650516 | X:55650821 | CDS | CDS | duplication |
|  |  |  |  |  |  |  |  |
| **CNS_NBL_6** | FOXR2 | FOXR2 | X:55650310 | X:55650622 | CDS | CDS | duplication |
| **CNS_NBL_6** | FOXR2 | TCF12 | X:55651256 | 15:57489970 | 3'UTR | splice-site | translocation/3'-3' |
|  |  |  |  |  |  |  |  |
| **CNS_NBL_7** | FOXR2 | FOXR2 | X:55650482 | X:55650921 | CDS | CDS | duplication |
|  |  |  |  |  |  |  |  |
| **CNS_NBL_8** | FOXR2 | FOXR2 | X:55650503 | X:55650590 | CDS | CDS | duplication |
|  |  |  |  |  |  |  |  |
| **CNS_NBL_9** | FOXR2 | FOXR2 | X:55650290 | X:55650799 | CDS | CDS | duplication |
|  |  |  |  |  |  |  |  |
| **CNS_NBL_10** | FOXR2 | FOXR2 | X:55650484 | X:55650878 | CDS | CDS | duplication |
|  |  |  |  |  |  |  |  |
| **CNS_NBL_11** | FOXR2 | FOXR2 | X:55650225 | X:55650634 | CDS | CDS | duplication |
| **CNS_NBL_11** | FOXR2 | FRMD4A | X:55650386 | 10:13956772 | CDS | intron | translocation/5'-5' |
|  |  |  |  |  |  |  |  |
| **CNS_NBL_12** | FOXR2 | FOXR2 | X:55650227 | X:55650854 | CDS | CDS | duplication |
|  |  |  |  |  |  |  |  |
| **CNS_NBL_13** | FOXR2 | FOXR2 | X:55650227 | X:55650993 | CDS | CDS | duplication |
|  |  |  |  |  |  |  |  |
| **CNS_NBL_14** | FOXR2 | FOXR2 | X:55650235 | X:55650865 | CDS | CDS | duplication |

**Supplementary Table1. Fusion variants in CNS neuroblastoma with forkhead box R2 (*FOXR2*) activation**

| **Case Number** | **Gene1** | **Gene2** | **Breakpoint_1** | **Breakpoint_2** | **Site_1** | **Site_2** | **Type** |
| --- | --- | --- | --- | --- | --- | --- | --- |
| **EPN_RELA_1** | C11orf95 | RELA | 11:63532340 | 11:65429676 | splice-site | splice-site | duplication |
| **EPN_RELA_2** | C11orf95 | RELA | 11:63532340 | 11:65429676 | splice-site | splice-site | duplication |
| **EPN_RELA_3** | C11orf95 | RELA | 11:63533279 | 11:65429676 | splice-site | splice-site | duplication |
| **EPN_RELA_4** | C11orf95 | RELA | 11:63532340 | 11:65429676 | splice-site | splice-site | duplication |
| **EPN_RELA_5** | C11orf95 | RELA | 11:63533279 | 11:65429676 | splice-site | splice-site | duplication |
| **EPN_RELA_6** | C11orf95 | RELA | 11:63532340 | 11:65429676 | splice-site | splice-site | duplication |
| **EPN_RELA_7** | C11orf95 | RELA | 11:63532340 | 11:65429676 | splice-site | splice-site | duplication |
| **EPN_RELA_8** | C11orf95 | RELA | 11:63532340 | 11:65429559 | splice-site | splice-site | duplication |
| **EPN_RELA_9** | C11orf95 | RELA | 11:63533279 | 11:65429676 | splice-site | splice-site | duplication |
| **EPN_RELA_10** | C11orf95 | RELA | 11:63533279 | 11:65429676 | splice-site | splice-site | duplication |
| **EPN_RELA_11** | C11orf95 | RELA | 11:63533279 | 11:65429676 | splice-site | splice-site | duplication |
| **EPN_RELA_12** | C11orf95 | RELA | 11:63532340 | 11:65429676 | splice-site | splice-site | duplication |
| **EPN_RELA_13** | C11orf95 | RELA | 11:63533279 | 11:65429676 | splice-site | splice-site | duplication |
| **EPN_RELA_14** | C11orf95 | RELA | 11:63533279 | 11:65429676 | splice-site | splice-site | duplication |
| **EPN_RELA_15** | C11orf95 | RELA | 11:63533279 | 11:65429676 | splice-site | splice-site | duplication |
| **EPN_RELA_16** | C11orf95 | RELA | 11:63532340 | 11:65429676 | splice-site | splice-site | duplication |
| **EPN_RELA_17** | C11orf95 | RELA | 11:63532340 | 11:65429559 | splice-site | splice-site | duplication |
| **EPN_RELA_18** | C11orf95 | RELA | 11:63533279 | 11:65429676 | splice-site | splice-site | duplication |
| **EPN_RELA_19** | C11orf95 | RELA | 11:63532340 | 11:65429676 | splice-site | splice-site | duplication |
| **EPN_RELA_20** | C11orf95 | RELA | 11:63533279 | 11:65429676 | splice-site | splice-site | duplication |

**Supplementary Table 2. Fusion variants in *RELA_C11orf95* ependymomas**

**Supplementary Table 3. List of 100 top genes overexpressed in CNS-NBL**

| **Gene** | **baseMean** | **log2FoldChange** | **pvalue** | **padj** |
| --- | --- | --- | --- | --- |
| **SOX10** | **894,678454** | **5,16308173** | **1,44E-68** | **7,03E-65** |
| DNM3 | 4405,88829 | 3,79126557 | 3,32E-89 | 4,86E-85 |
| KCNS3 | 111,211499 | 4,30686238 | 3,74E-61 | 7,31E-58 |
| **ANKRD55** | **138,130955** | **5,68590472** | **4,07E-59** | **7,46E-56** |
| PFN2 | 2114,70946 | 2,66304543 | 3,29E-50 | 3,71E-47 |
| RBPJ | 2758,1462 | 1,44446077 | 1,09E-48 | 1,06E-45 |
| ATP6V1A | 1729,40886 | 1,78615897 | 1,67E-48 | 1,58E-45 |
| RGMB | 1421,60111 | 4,36699827 | 2,11E-48 | 1,94E-45 |
| CYP4X1 | 70,076825 | 4,17306314 | 1,98E-42 | 1,41E-39 |
| RNF150 | 691,60982 | 2,96673719 | 2,83E-41 | 1,85E-38 |
| MFAP3 | 395,060501 | 1,26839139 | 6,73E-38 | 3,65E-35 |
| NREP | 1417,93317 | 2,73879459 | 4,88E-36 | 2,33E-33 |
| GREM1 | 92,7281439 | 7,4677333 | 4,92E-36 | 2,33E-33 |
| FGF14 | 653,683124 | 5,52400968 | 5,10E-36 | 2,37E-33 |
| FOXR2 | 48,3447316 | 9,00670435 | 6,47E-36 | 2,97E-33 |
| MRPS23 | 279,670819 | 1,71914445 | 1,72E-35 | 7,66E-33 |
| FABP6 | 53,777866 | 5,91454714 | 1,93E-35 | 8,46E-33 |
| SMARCD1 | 1576,16984 | 1,41843926 | 3,26E-35 | 1,39E-32 |
| TOMM20 | 1213,56349 | 2,10710746 | 1,82E-34 | 7,40E-32 |
| BMP8B | 65,5693438 | 2,94169277 | 1,85E-33 | 6,96E-31 |
| COX7A2 | 895,808462 | 1,78900849 | 3,73E-33 | 1,33E-30 |
| ATAT1 | 1422,03872 | 1,73078685 | 8,36E-33 | 2,92E-30 |
| GSTA4 | 931,032372 | 1,3693643 | 4,66E-32 | 1,52E-29 |
| ATP8A2 | 856,150486 | 5,13437338 | 5,82E-32 | 1,88E-29 |
| EVPL | 33,9061183 | 4,13861673 | 6,08E-32 | 1,94E-29 |
| SCAMP5 | 366,727735 | 2,1055131 | 1,74E-31 | 5,43E-29 |
| RAB3A | 408,5629 | 3,48536203 | 3,96E-31 | 1,17E-28 |
| KCNJ9 | 39,9855819 | 4,28273905 | 5,47E-31 | 1,56E-28 |
| KIAA1045 | 53,2186059 | 4,48984658 | 5,47E-31 | 1,56E-28 |
| NDRG3 | 1146,91385 | 1,46684138 | 8,18E-31 | 2,29E-28 |
| POLR3D | 643,760001 | 1,79864616 | 1,21E-30 | 3,35E-28 |
| SHISA7 | 50,4897389 | 5,08200424 | 2,11E-30 | 5,63E-28 |
| FAM32A | 488,539306 | 1,5399864 | 3,00E-30 | 7,92E-28 |
| SMAP2 | 1007,64502 | 2,19780993 | 4,31E-30 | 1,11E-27 |
| UBL7 | 647,748683 | 1,6870147 | 5,99E-30 | 1,53E-27 |
| GAL3ST1 | 47,6937198 | 3,95315133 | 7,33E-30 | 1,85E-27 |
| CHRNB2 | 843,896199 | 3,71481356 | 2,38E-29 | 5,75E-27 |
| MATN4 | 46,8765531 | 6,63969355 | 2,39E-29 | 5,75E-27 |
| TSG101 | 1465,35415 | 1,40633541 | 3,96E-29 | 9,44E-27 |
| HMGB3P7 | 4,85361212 | 5,41976951 | 1,02E-28 | 2,37E-26 |
| LIN7C | 1046,02166 | 1,44466579 | 1,94E-28 | 4,37E-26 |
| RASGRF1 | 236,450701 | 5,48722985 | 2,10E-28 | 4,70E-26 |
| DPYSL3 | 6072,57537 | 2,52625268 | 2,19E-28 | 4,87E-26 |
| RGR | 462,816151 | 4,44183089 | 2,31E-28 | 5,10E-26 |
| KIT | 1027,61859 | 3,70779279 | 2,68E-28 | 5,86E-26 |
| GLRA3 | 377,591703 | 9,38046287 | 2,81E-28 | 6,06E-26 |
| TOMM70A | 1053,23221 | 1,65175649 | 2,96E-28 | 6,34E-26 |
| HCAR1 | 53,6339796 | 4,68635173 | 3,55E-28 | 7,49E-26 |
| ATP6V1G2 | 112,660721 | 2,69174546 | 5,09E-28 | 1,07E-25 |
| RP11-167P23.4 | 7,28027391 | 6,96630698 | 6,20E-28 | 1,29E-25 |
| GDAP1L1 | 279,338083 | 3,24655634 | 8,36E-28 | 1,70E-25 |
| C1QTNF4 | 30,551402 | 4,1282919 | 1,16E-27 | 2,29E-25 |
| MMP24 | 461,394782 | 2,50981732 | 1,92E-27 | 3,78E-25 |
| METTL2A | 698,980989 | 1,20905775 | 2,95E-27 | 5,72E-25 |
| RELL2 | 21,6382924 | 2,03236931 | 4,45E-27 | 8,38E-25 |
| NXPH1 | 538,898293 | 6,85216703 | 5,38E-27 | 9,99E-25 |
| KCNIP2 | 183,826585 | 2,96347299 | 5,90E-27 | 1,07E-24 |
| PRKG2 | 490,0475 | 5,90586871 | 1,03E-26 | 1,84E-24 |
| FBLL1 | 87,5388192 | 3,48118608 | 1,05E-26 | 1,88E-24 |
| COL9A2 | 1302,07796 | 3,16746575 | 1,11E-26 | 1,97E-24 |
| PFDN2 | 474,627373 | 2,10825007 | 1,46E-26 | 2,48E-24 |
| LANCL1 | 1137,83633 | 1,51404176 | 1,91E-26 | 3,23E-24 |
| NCAM1 | 8966,20478 | 2,27826346 | 2,54E-26 | 4,23E-24 |
| NLGN1 | 1968,41659 | 2,3814652 | 3,23E-26 | 5,32E-24 |
| SPHKAP | 585,03064 | 5,44014417 | 7,57E-26 | 1,21E-23 |
| GDAP1 | 5381,59737 | 1,97654566 | 8,67E-26 | 1,37E-23 |
| PAFAH1B1 | 4575,60609 | 0,99793422 | 1,08E-25 | 1,69E-23 |
| ADAMTS20 | 453,759625 | 6,27116355 | 1,09E-25 | 1,70E-23 |
| STS | 386,767095 | 1,77503212 | 1,12E-25 | 1,73E-23 |
| AKR1E2 | 97,5240995 | 3,36296882 | 1,12E-25 | 1,73E-23 |
| IQSEC3 | 79,581899 | 4,13860804 | 1,88E-25 | 2,84E-23 |
| SYN1 | 737,783003 | 3,70943806 | 2,11E-25 | 3,15E-23 |
| TBC1D30 | 679,569424 | 3,03569371 | 2,83E-25 | 4,17E-23 |
| FAM184B | 759,315013 | 3,28488159 | 4,38E-25 | 6,39E-23 |
| NDUFB9 | 1014,82235 | 1,48435383 | 5,05E-25 | 7,26E-23 |
| FBXO45 | 103,643572 | 1,40698166 | 7,29E-25 | 1,03E-22 |
| KLRC2 | 858,667067 | 7,54281113 | 7,38E-25 | 1,04E-22 |
| SMTNL2 | 14,2250001 | 4,40225488 | 8,62E-25 | 1,19E-22 |
| LEPROTL1 | 208,923558 | 1,37983675 | 1,08E-24 | 1,47E-22 |
| CAMK2N2 | 37,1656254 | 3,63556987 | 1,31E-24 | 1,76E-22 |
| EIF2B1 | 1727,40625 | 1,05947748 | 1,33E-24 | 1,77E-22 |
| SSR2 | 2076,98367 | 2,02422873 | 1,41E-24 | 1,88E-22 |
| EPCAM | 227,787053 | 4,4358999 | 1,79E-24 | 2,35E-22 |
| TMEM33 | 1160,51291 | 1,32548967 | 2,22E-24 | 2,89E-22 |
| MVB12B | 1259,13826 | 1,94406138 | 2,67E-24 | 3,43E-22 |
| GABRB3 | 1496,58833 | 4,72272158 | 2,84E-24 | 3,64E-22 |
| SPPL3 | 1068,79443 | 1,00878857 | 4,64E-24 | 5,84E-22 |
| CXADR | 557,483958 | 2,89581572 | 5,42E-24 | 6,73E-22 |
| TNR | 3046,13017 | 5,78648686 | 7,02E-24 | 8,69E-22 |
| C1QL1 | 1491,22447 | 3,74020315 | 1,08E-23 | 1,30E-21 |
| SV2A | 918,052664 | 2,31451177 | 1,45E-23 | 1,73E-21 |
| C17orf58 | 55,9615975 | 1,53612786 | 3,11E-23 | 3,62E-21 |
| WDR46 | 778,778166 | 1,27911979 | 3,41E-23 | 3,92E-21 |
| SH3BP5L | 424,271523 | 1,27320509 | 6,11E-23 | 6,92E-21 |
| KCNAB1 | 267,19612 | 2,77155034 | 7,57E-23 | 8,42E-21 |
| ZNF648 | 60,6327429 | 4,70707155 | 9,63E-23 | 1,06E-20 |
| RPAIN | 1129,03824 | 1,18190282 | 1,01E-22 | 1,11E-20 |
| TOMM22 | 384,390829 | 1,31591811 | 1,01E-22 | 1,11E-20 |
| DERL1 | 482,897637 | 1,35287254 | 1,15E-22 | 1,25E-20 |
| PSMD13 | 1302,93999 | 1,02782176 | 1,51E-22 | 1,60E-20 |

**Supplementary Table 4. Signaling pathways activated in various CNS-PNET cohorts**

| **GOID** | **GO-Term** | **Ontology Source** | **Term P-Value** | **Group P-Value** | **Associated Genes Found** |
| --- | --- | --- | --- | --- | --- |
| GO:0004721 | Synaptic vesicle cycle | KEGG_10.02.2016 | 480,0E-6 | 480,0E-6 | ATP6V1G2, DNM3, RAB3A, SNAP25, STXBP1 |
| GO:0004911 | Insulin secretion | KEGG_10.02.2016 | 47,0E-3 | 47,0E-3 | KCNMA1, RAB3A, RAPGEF4, SNAP25 |
| GO:0004520 | Adherens junction | KEGG_10.02.2016 | 110,0E-3 | 110,0E-3 | PTPRJ, TCF7L2, WASF1 |
| GO:0000240 | Pyrimidine metabolism | KEGG_10.02.2016 | 26,0E-3 | 94,0E-3 | NME1, NT5C3B, POLR1D, POLR2F, POLR3D |
| GO:0003020 | RNA polymerase | KEGG_10.02.2016 | 14,0E-3 | 94,0E-3 | POLR1D, POLR2F, POLR3D |
| GO:0000190 | Oxidative phosphorylation | KEGG_10.02.2016 | 1,2E-6 | 2,3E-3 | ATP5J, ATP6V1A, COX6A1, COX6B1, COX7A2, |
| GO:0005010 | Alzheimer's disease | KEGG_10.02.2016 | 1,4E-3 | 2,3E-3 | ATP5H, ATP5J, COX6A1, COX6B1, COX7A2, |
| GO:0005016 | Huntington's disease | KEGG_10.02.2016 | 1,0E-3 | 2,3E-3 | NDUFB9, NDUFS2, POLR2F, UQCRH, UQCRQ |
| GO:0003008 | Ribosome biogenesis | KEGG_10.02.2016 | 54,0E-3 | 54,0E-3 | NHP2, RIOK1, SNU13, WDR3 |
| GO:0003010 | Ribosome | KEGG_10.02.2016 | 22,0E-3 | 22,0E-3 | MRPS21, RPL30, RPL37, RPL38, RPS21, RSL24D1 |
| GO:0003050 | Proteasome | KEGG_10.02.2016 | 33,0E-3 | 33,0E-3 | PSMB4, PSMD13, PSME3 |
| GO:0004512 | ECM-receptor interaction | KEGG_10.02.2016 | 42,0E-3 | 42,0E-3 | COL9A1, COL9A2, SV2A, TNR |

**CNS neuroblastoma with forkhead box R2 (*FOXR2*) activation**

| **GOID** | **GOTerm** | **Ontology Source** | **Term PValue** | **Group PValue** | **Associated Genes Found** |
| --- | --- | --- | --- | --- | --- |
| GO:0000310 | Lysine degradation | KEGG_10.02.2016 | 13,0E-3 | 13,0E-3 | DOT1L, KMT2C, PIPOX |
| GO:0003460 | Fanconi anemia pathway | KEGG_10.02.2016 | 14,0E-3 | 14,0E-3 | BRCA1, FANCD2, REV3L |
| GO:0004068 | FoxO signaling pathway | KEGG_10.02.2016 | 8,9E-3 | 8,9E-3 | CCNB3, FOXG1, FOXO1, HOMER3 |
| GO:0004611 | Platelet activation | KEGG_10.02.2016 | 29,0E-3 | 29,0E-3 | GP1BA, GP6, MAPK1, TLN2 |
| GO:0005215 | Prostate cancer | KEGG_10.02.2016 | 53,0E-3 | 53,0E-3 | FOXO1, MAPK1, PDGFRA |

**Glioblastoma IDH wild-type subclass H3.3 G34**

| **GOID** | **GOTerm** | **Ontology Source** | **Term PValue** | **Group PValue** | **Associated Genes Found** |
| --- | --- | --- | --- | --- | --- |
| GO:0003008 | Ribosome biogenesis | KEGG_10.02.2016 | 150,0E-6 | 150,0E-6 | DKC1, FBL, GNL3, MPHOSPH10, |
| GO:0003013 | RNA transport | KEGG_10.02.2016 | 79,0E-6 | 79,0E-6 | DDX20, NUP205, NUPL2, PABPC4, |
| GO:0003015 | mRNA surveillance pathway | KEGG_10.02.2016 | 110,0E-3 | 110,0E-3 | CSTF1, PABPC4, SMG1 |
| GO:0003018 | RNA degradation | KEGG_10.02.2016 | 3,3E-3 | 3,3E-3 | HSPD1, PABPC4, PARN, PNPT1, ZCCHC7 |
| GO:0004110 | Cell cycle | KEGG_10.02.2016 | 23,0E-3 | 23,0E-3 | ATR, DBF4, ORC2, ORC5, SKP2 |
| GO:0004146 | Peroxisome | KEGG_10.02.2016 | 95,0E-3 | 95,0E-3 | IDH1, NUDT19, PEX13 |
| GO:0004912 | GnRH signaling pathway | KEGG_10.02.2016 | 110,0E-3 | 28,0E-3 | CAMK2D, ITPR1, SOS1 |
| GO:0004922 | Glucagon signaling pathway | KEGG_10.02.2016 | 10,0E-3 | 28,0E-3 | CAMK2D, ITPR1, PHKA1, PPP4R3B, PYGL |

**Glioblastoma IDH wild-type subclass MYCN**

| **GOID** | **GOTerm** | **Ontology Source** | **Term PValue** | **Group PValue** | **Associated Genes Found** |
| --- | --- | --- | --- | --- | --- |
| GO:0000562 | Inositol phosphate metabolism | KEGG_10.02.2016 | 18,0E-3 | 18,0E-3 | INPP5A, ITPKC, PLCH2 |
| GO:0004310 | Wnt signaling pathway | KEGG_10.02.2016 | 810,0E-6 | 5,8E-3 | AXIN2, NFATC1, NKD2, WNT3A, WNT7B |
| GO:0004340 | Hedgehog signaling pathway | KEGG_10.02.2016 | 7,3E-3 | 5,8E-3 | PRKACA, WNT3A, WNT7B |
| GO:0005217 | Basal cell carcinoma | KEGG_10.02.2016 | 9,0E-3 | 5,8E-3 | AXIN2, WNT3A, WNT7B |
| GO:0004066 | HIF-1 signaling pathway | KEGG_10.02.2016 | 8,5E-3 | 8,5E-3 | ERBB2, MKNK2, PFKL, RELA |
| GO:0004010 | MAPK signaling pathway | KEGG_10.02.2016 | 770,0E-6 | 1,2E-3 | MAPKAPK3, NFATC1, PRKACA, RELA |
| GO:0004020 | Calcium signaling pathway | KEGG_10.02.2016 | 2,7E-3 | 1,2E-3 | CACNA1I, ERBB2, ITPKC, P2RX5, |
| GO:0004925 | Aldosterone synthesis and secretion | KEGG_10.02.2016 | 25,0E-3 | 1,2E-3 | CACNA1H, CACNA1I, PRKACA |
| GO:0004974 | Protein digestion and absorption | KEGG_10.02.2016 | 5,3E-3 | 5,3E-3 | COL18A1, COL6A1, ELN, SLC9A3 |

**Ependymoma with *RELA_C11orf95* fusion**

**Supplementary Table 5. Top 100 genes differentially expressed between CNS_NBL and GBM_MID**

| **Gene** | **baseMean** | **log2FoldChange** | **pvalue** | **padj** |
| --- | --- | --- | --- | --- |
| OBSCN | 15488,3039 | 3,98344692 | 1,40E-42 | 3,87E-38 |
| **ANKRD55** | **312,388912** | **4,36759823** | **2,58E-40** | **3,55E-36** |
| GPC3 | 5547,58121 | 7,05670232 | 1,76E-31 | 1,61E-27 |
| NFIB | 10762,0366 | 2,39584398 | 3,08E-31 | 2,12E-27 |
| WIF1 | 946,438486 | 7,54933647 | 5,47E-30 | 3,01E-26 |
| DLX6 | 823,429367 | 7,63986082 | 1,42E-29 | 6,53E-26 |
| GPR12 | 587,761704 | 4,69881252 | 6,29E-28 | 2,47E-24 |
| NKX2-1 | 353,087463 | 7,9696024 | 3,56E-27 | 1,22E-23 |
| POLN | 333,349277 | -2,49611073 | 2,42E-26 | 6,67E-23 |
| COCH | 486,593614 | 2,89153928 | 2,24E-26 | 6,67E-23 |
| DGKB | 9993,97554 | 4,85263264 | 4,31E-26 | 9,90E-23 |
| TBC1D30 | 1267,25342 | 3,03304065 | 4,24E-26 | 9,90E-23 |
| KCNA5 | 683,727811 | 6,72880843 | 6,56E-26 | 1,39E-22 |
| FAM163A | 144,775392 | 7,50033401 | 4,93E-25 | 9,04E-22 |
| HCAR1 | 113,482254 | 3,66706558 | 4,96E-25 | 9,04E-22 |
| BCL11B | 240,664521 | 3,63143977 | 5,25E-25 | 9,04E-22 |
| RPS21 | 713,460499 | 2,52619083 | 1,10E-24 | 1,77E-21 |
| CHRNB2 | 1603,32561 | 4,15775482 | 2,19E-24 | 3,35E-21 |
| TENM2 | 1595,2002 | 3,77782893 | 1,92E-23 | 2,79E-20 |
| TSHR | 5567,71131 | 4,5132211 | 4,55E-23 | 6,26E-20 |
| TENM3 | 4234,01031 | 4,89930784 | 6,98E-23 | 9,16E-20 |
| CDH8 | 1429,13036 | 4,69195418 | 8,47E-23 | 1,06E-19 |
| ANKRD13B | 1111,79403 | 1,66655585 | 3,04E-22 | 3,64E-19 |
| WNK2 | 3509,49098 | 3,21280367 | 1,55E-21 | 1,77E-18 |
| RPSAP63 | 67,1685996 | 7,3145471 | 4,54E-21 | 5,00E-18 |
| POLR1E | 956,791773 | 1,50499002 | 6,61E-21 | 7,00E-18 |
| SLC6A9 | 226,967964 | -2,81274015 | 6,88E-21 | 7,02E-18 |
| PTGIS | 292,460366 | 2,72894362 | 7,36E-21 | 7,24E-18 |
| COL2A1 | 1574,36225 | 5,05266325 | 1,24E-20 | 1,18E-17 |
| ELAVL2 | 1203,60222 | 3,88363909 | 1,51E-20 | 1,39E-17 |
| FAM71F2 | 136,244202 | -1,97868745 | 2,00E-20 | 1,78E-17 |
| STXBP3 | 1438,25283 | -1,38350122 | 2,17E-20 | 1,83E-17 |
| SMTNL2 | 28,2024442 | 5,10764293 | 2,20E-20 | 1,83E-17 |
| KAT2B | 2896,90647 | -2,71529636 | 2,48E-20 | 2,01E-17 |
| MTA1 | 1840,08305 | 1,31817102 | 3,42E-20 | 2,69E-17 |
| TCF4 | 26606,5243 | 1,93971086 | 3,94E-20 | 3,01E-17 |
| RPL18 | 6763,05453 | 2,26711206 | 5,99E-20 | 4,46E-17 |
| EIF4EBP1 | 360,961337 | 2,43820247 | 8,55E-20 | 6,20E-17 |
| C5orf64 | 31,0394155 | -3,96244771 | 1,53E-19 | 1,07E-16 |
| FOXR2 | 107,010027 | 7,46540029 | 1,56E-19 | 1,07E-16 |
| DLX5 | 981,582506 | 6,00615784 | 1,68E-19 | 1,13E-16 |
| HRH3 | 119,709975 | 5,1378247 | 2,36E-19 | 1,55E-16 |
| RNF150 | 1249,37285 | 2,67105875 | 4,97E-19 | 3,19E-16 |
| CELF3 | 1803,63116 | 3,09025295 | 6,49E-19 | 4,06E-16 |
| MEGF6 | 169,161622 | 3,68248152 | 7,46E-19 | 4,50E-16 |
| SH3GL1P3 | 71,4069116 | 2,06697953 | 7,52E-19 | 4,50E-16 |
| CDHR1 | 1969,70966 | 3,03201663 | 8,17E-19 | 4,78E-16 |
| PDGFC | 341,986617 | -2,931323 | 9,84E-19 | 5,64E-16 |
| NHP2 | 933,279286 | 1,55063283 | 1,29E-18 | 7,25E-16 |
| PTGR1 | 420,668438 | -1,71465742 | 1,39E-18 | 7,66E-16 |
| HIST1H2BF | 1939,82601 | 3,76068725 | 1,53E-18 | 8,28E-16 |
| TSC2 | 8947,65272 | 1,40255301 | 3,10E-18 | 1,64E-15 |
| CYB5R2 | 128,908932 | -3,44392817 | 3,29E-18 | 1,71E-15 |
| SLC32A1 | 180,384075 | 5,12689522 | 3,69E-18 | 1,88E-15 |
| IQCA1 | 183,754101 | -2,82602178 | 4,64E-18 | 2,28E-15 |
| USP27X | 142,859825 | 2,11951641 | 4,59E-18 | 2,28E-15 |
| AP3B2 | 1688,80541 | 2,1404522 | 5,45E-18 | 2,63E-15 |
| TFB2M | 676,32413 | 1,75229696 | 6,62E-18 | 3,14E-15 |
| TTC18 | 237,611437 | -3,45940005 | 7,75E-18 | 3,62E-15 |
| TFR2 | 315,328747 | 2,31105837 | 9,40E-18 | 4,32E-15 |
| PTPN12 | 3806,35288 | -1,23099521 | 1,33E-17 | 5,99E-15 |
| INPPL1 | 1487,75717 | -2,20463029 | 1,47E-17 | 6,55E-15 |
| GLRA3 | 844,595186 | 5,64765803 | 2,12E-17 | 9,25E-15 |
| HIST3H2A | 709,987119 | 4,01976681 | 3,20E-17 | 1,37E-14 |
| FAXC | 534,920096 | 1,96850607 | 3,22E-17 | 1,37E-14 |
| RGMB | 2930,93153 | 3,73765387 | 4,82E-17 | 2,01E-14 |
| MST4 | 209,083568 | 3,61748725 | 6,33E-17 | 2,60E-14 |
| GDAP1L1 | 539,061715 | 2,6325961 | 9,22E-17 | 3,73E-14 |
| REST | 733,212491 | -3,03987717 | 9,85E-17 | 3,93E-14 |
| RPS5 | 6610,35172 | 2,34505251 | 1,05E-16 | 4,15E-14 |
| CECR7 | 81,17765 | 2,81369183 | 1,11E-16 | 4,32E-14 |
| EFCAB14 | 1183,79476 | -1,70757252 | 1,27E-16 | 4,87E-14 |
| TSPAN2 | 95,9756384 | 2,59836625 | 1,35E-16 | 5,10E-14 |
| RAB3IP | 5868,88534 | 1,93904267 | 1,49E-16 | 5,53E-14 |
| EFNA5 | 229,215809 | 4,21811014 | 2,14E-16 | 7,84E-14 |
| NIPSNAP1 | 1668,86675 | 2,19373429 | 2,78E-16 | 1,01E-13 |
| FAM69A | 359,580885 | -1,89886043 | 2,87E-16 | 1,03E-13 |
| ZSWIM5 | 635,60969 | 1,39260625 | 3,34E-16 | 1,18E-13 |
| ADAM9 | 10361,949 | -2,28746614 | 3,69E-16 | 1,29E-13 |
| SMAD9 | 1225,13311 | 2,11839191 | 4,00E-16 | 1,38E-13 |
| TOP1MT | 420,769868 | 1,37369717 | 4,19E-16 | 1,41E-13 |
| SCD | 1858,36812 | -2,00608209 | 4,21E-16 | 1,41E-13 |
| CUX2 | 933,520915 | 4,43478583 | 4,80E-16 | 1,59E-13 |
| RPS11 | 24195,954 | 1,92102207 | 5,89E-16 | 1,93E-13 |
| ADCYAP1 | 104,040414 | 6,44718822 | 7,04E-16 | 2,28E-13 |
| LGR5 | 2674,39572 | 3,66827385 | 7,29E-16 | 2,33E-13 |
| CNST | 1010,29862 | 1,19857751 | 8,10E-16 | 2,56E-13 |
| CACNA1E | 3702,04106 | 3,15182265 | 9,36E-16 | 2,93E-13 |
| LEPROT | 136,315578 | -1,65082782 | 9,73E-16 | 3,01E-13 |
| CDK6 | 382,099847 | -3,58532586 | 1,14E-15 | 3,50E-13 |
| GPSM1 | 853,593483 | 1,37900006 | 1,20E-15 | 3,62E-13 |
| RAC3 | 378,987723 | 2,38138296 | 1,29E-15 | 3,88E-13 |
| REEP3 | 982,880346 | -1,37795038 | 1,43E-15 | 4,22E-13 |
| ENDOD1 | 472,897794 | -2,07599137 | 1,63E-15 | 4,76E-13 |
| RPL19 | 17989,0364 | 1,78851831 | 1,75E-15 | 5,07E-13 |
| AASS | 3033,76899 | -2,79768682 | 1,99E-15 | 5,72E-13 |
| NT5E | 1089,23748 | -3,42154683 | 2,12E-15 | 6,02E-13 |
| TMEM87B | 568,132299 | -1,56484916 | 2,34E-15 | 6,51E-13 |
| GEMIN4 | 571,34696 | 1,38784177 | 2,34E-15 | 6,51E-13 |
| CPM | 359,860111 | -1,66534468 | 2,44E-15 | 6,72E-13 |
