# Supplementary material for: Molecular analysis of pediatric CNS-PNET revealed nosologic heterogeneity and potent diagnostic markers for CNS neuroblastoma with FOXR2-activation
Source: Acta Neuropathol Commun. 2021 Feb 3;9:20. doi: 10.1186/s40478-021-01118-5 (PMC7860633; doi:10.1186/s40478-021-01118-5)
Supplement: Supplementary file 2 — Additional file 2. [file 40478_2021_1118_MOESM2_ESM.docx]

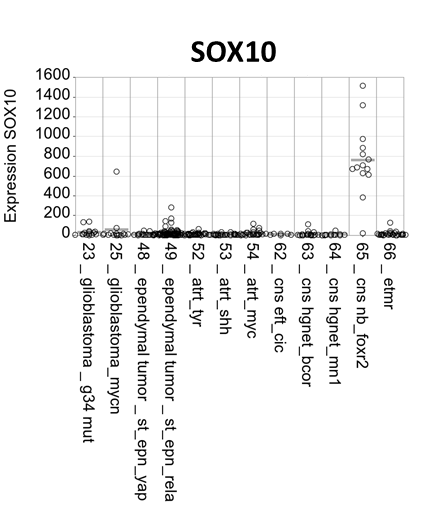


**Supplementary Figure 1. Expression of SOX-10 in various CNS tumors detected by Affymetrix platform.**
